# Association between serum levels of caspase-cleaved cytokeratin-18 and early mortality in patients with severe spontaneous intracerebral hemorrhage

**DOI:** 10.1186/s12868-018-0424-1

**Published:** 2018-04-16

**Authors:** Leonardo Lorente, María M. Martín, Antonia Pérez-Cejas, Luis Ramos, Mónica Argueso, Jordi Solé-Violán, Juan J. Cáceres, Alejandro Jiménez, Victor García-Marín

**Affiliations:** 1 Intensive Care Unit, Hospital Universitario de Canarias, Ofra, s/n, La Laguna, 38320 Santa Cruz de Tenerife, Spain; 2 Intensive Care Unit, Hospital Universitario Nuestra Señora de Candelaria, Crta del Rosario s/n, 38010 Santa Cruz de Tenerife, Spain; 3 Laboratory Department, Hospital Universitario de Canarias, Ofra, s/n, La Laguna, 38320 Santa Cruz de Tenerife, Spain; 4 Intensive Care Unit, Hospital General La Palma, Buenavista de Arriba s/n, Breña Alta, 38713 La Palma, Spain; 5 Intensive Care Unit, Hospital Clínico Universitario de Valencia, Avda. Blasco Ibáñez no 17-19, 46004 Valencia, Spain; 6 Intensive Care Unit, Hospital Universitario Dr. Negrín, CIBERES, Barranco de la Ballena s/n, 35010 Las Palmas de Gran Canaria, Spain; 7 Intensive Care Unit, Hospital Insular, Plaza Dr. Pasteur s/n, 35016 Las Palmas de Gran Canaria, Spain; 8 Research Unit, Hospital Universitario de Canarias, Ofra, s/n, La Laguna, 38320 Santa Cruz de Tenerife, Spain; 9 Department of Neurosurgery, Hospital Universitario de Canarias, Ofra, s/n, La Laguna, 38320 Santa Cruz de Tenerife, Spain

**Keywords:** Caspase-cleaved cytokeratin-18, Spontaneous intracerebral hemorrhage, Patients, Mortality

## Abstract

**Background:**

Apoptotic changes after cerebral hemorrhage in brain samples of humans have been found. Caspase-cleaved cytokeratin (CCCK)-18 could be detected in the bloodstream during apoptosis. Higher circulating CCCK-18 levels have been associated with 6-month mortality in patients with basal ganglia hemorrhage. The aim of our study was to determine whether there is an association between serum CCCK-18 levels and early mortality of spontaneous intracerebral hemorrhage (SIH) patients. We performed an observational, prospective and multicentre study. There were included patients with severe SIH defined as Glasgow Coma Scale (GCS) lower than 9. We determined serum CCCK-18 levels at the severe SIH diagnosis moment.

**Results:**

We found that non-surviving SIH patients (n = 46) showed lower GCS, and higher serum CCCK-18 levels and APACHE-II score than survivor ones (n = 54). In ROC analysis was found that the area under the curve of serum CCCK-18 levels for 30-day mortality prediction was 90% (95% CI 82–95%; p < 0.001). In the multiple logistic regression analysis, we found an association between serum CCCK-18 levels and 30-day mortality (OR 1.034; 95% CI 1.013–1.055; p = 0.002).

**Conclusions:**

The novel finding of our study was that there is an association between high serum CCCK-18 levels and 30-day mortality in severe SIH patients.

## Introduction

Spontaneous intracerebral hemorrhage (SIH) results in a large amount of deaths, disabilities, and resource consumption [1–3]. Cell death by apoptosis occurs in cerebral hemorrhage [4–7]. Primary damage is caused by the effect of hematoma leading to disruption and mechanical deformation of cellular architecture. Secondary damage is induced by mitochondrial dysfunction, microglia activation and the release of neurotransmitter and inflammatory mediators; and those events lead to necrosis and to the occurrence of programmed cell death by apoptosis [4–7].

Between 1999 and 2005 in some animals models were found the presence of apoptosis after cerebral hemorrhage [8–11]. In those studies with rats and rabbits using the terminal deoxynucleotidyl transferase (TdT)-mediated deoxyuridine (dUTP)-biotin nick end labeling (TUNEL) were found cells undergoing DNA fragmentation in the striatum and in deep white matter in the frontal lobe; and those cells were mostly neurons and astrocytes in the center and periphery of cerebral hemorrhage [8–11]. Afterwards, apoptotic changes after cerebral hemorrhage in human brain samples have been found in several studies from 2003, and included TUNEL-positive cells and an increase of caspase-3 expression [12–18].

Cytokeratins (CK) family is a group of proteins distributed mainly in the epithelial tissue. Until now are known 20 types, named CK-1 to CK-20. CK-18 is cleaved by the action of caspases during apoptosis, then caspase-cleaved cytokeratin (CCCK)-18 appears and could be detected in the bloodstream [19, 20].

Higher circulating CCCK-18 levels have been found in patients with sepsis [21–25], liver diseases [26–30], and tumoral diseases [31, 32]. Besides, higher circulating CCCK-18 levels have been associated with a poor prognosis in patients with cerebral process, such as traumatic brain injury [33], aneurysmal subarachnoid hemorrhage [34] and basal ganglia hemorrage [35]. In the study by Gu et al. of patients with basal ganglia hemorrage was found an association between circulating CCCK-18 levels and 6-month mortality [35]. The aim of our study was to determine whether there is an association between serum CCCK-18 levels and early mortality of SIH patients.

## Methods

### Design and subjects

This multicentre, observational and prospective study was carried in six Intensive Care Units from Spain after the Institutional Review Board approval of all participating hospitals and with the written consent of patient guardians. Participating hospitals were the following: H. Clínico Universitario de Valencia (Valencia), H. Universitario Nuestra Señora de Candelaria (Tenerife), H. General de La Palma (La Palma), H. Universitario de Canarias (Tenerife), H. Insular (Gran Canaria), H. Universitario Dr. Negrín (Gran Canaria).

We included patients with severe supratentorial SIH. We used Glasgow Coma Scale (GCS) [36] to classify SIH severity; and we considered severe SIH when GCS ≤ 8. Exclusion criteria were infratentorial or traumatic hemorrhage, inflammatory or malignant disease, hemorrhagic transformation of cerebral infarction, age < 18 years, and pregnancy.

### Variables recorded

The following variables were recorded for each patient: sex, site and cause of SIH, volume of SIH, presence of intraventricular hemorrhage or hydrocephalus, midline shift, evacuation of SIH, age, sodium, temperature, platelets, pressure of arterial oxygen (paO2), fraction of inspired oxygen (FIO2), international normalised ratio (INR), fibrinogen, activated partial thromboplastin time (aPTT), lactic acid, GCS, creatinine, glycaemia, Acute Physiology and Chronic Health Evaluation II (APACHE II) score [37]. The end-point study was mortality at 30 days after severe SIH diagnosis.

### Blood sample collection and serum CCCK-18 analysis

We collected serum blood samples when severe SIH was diagnosed to determine serum CCCK-18 levels. We determined serum CCCK-18 levels at the Laboratory Department of the Hospital Universitario de Canarias (Tenerife, Spain) by an enzyme-linked immunosorbent assay (ELISA). The kit used was M30 Apoptosense^®^ ELISA kit (PEVIVA AB, Bromma, Sweden), and its intra-assay coefficient of variation (CV), inter-assay CV, and detection limit assay were < 10, < 10% and 25 u/L, respectively.

### Statistical methods

We reported continuous and categorical variables as medians (with interquartile ranges) and frequencies (with percentages) respectively. We compared continuous and categorical variables between groups by means of Wilcoxon–Mann–Whitney test and Chi square test respectively. We used multiple logistic regression analysis to determine the association of serum CCCK-18 levels with 30-day mortality, after controlling for GCS, midline shift, age, intraventricular hemorrhage, and volume of intracerebral hemorrhage. Odds Ratio and its 95% confidence intervals (CI) were calculated to estimate the impact of each predictor variable. A receiver operating characteristic (ROC) analysis was carried out to determine the capacity of 30 day-mortality prediction by serum CCCK-18 levels. We made 30 day-mortality Kaplan–Meier curves of SIH patients with higher/lower serum CCCK-18 levels than 156 u/L (due to that those serum CCCK-18 levels were the optimal prognostic cut-off, for sensitivity and specificity, according to Youden J index). Each *p* value lower than 0.05 was considered as statistically significant. We performed statistical analyses by SPSS 17.0 (SPSS Inc., Chicago, IL, USA), LogXact 4.1, (Cytel Co., Cambridge, MA), and NCSS 2000 (Kaysville, Utah).

## Results

Of the 100 patients with severe SIH, a total of 54 patients were alive at 30 days and 46 patients died during the first 30 days of SIH diagnosis. We did not find statistically significant differences between patient groups (non-surviving and surviving) in sex, site of SIH, cause of SIH, intraventricular hemorrhage, hydrocephalus, SIH evacuation, sodium, temperature, sodium, PaO2/FI02 ratio, platelets, INR, lactic acid, fibrinogen, creatinine, and aPTT. However, we found that non-surviving SIH patients showed lower GCS, and higher serum CCCK-18 levels, APACHE-II score, age, volume of SIH, midline shift, and glycemia than survivor ones (Table 1).

**Table 1:** Clinical and biochemical characteristics of 30-day surviving and non-surviving patients with spontaneous intracerebral hemorrhage (SIH)

	Survivors (n = 54)	Non-survivors (n = 46)	P value
Gender female—n (%)	17 (31.5)	17 (37.0)	0.67
Site of SIH—n (%)			0.81
Lobar	41 (75.9)	33 (71.7)	
Basal ganglia	3 (5.6)	4 (8.7)	
Thalamus	5 (9.3)	3 (6.5)	
Periventricular	5 (9.3)	6 (13.0)	
Cause of SIH—n (%)			0.07
Hypertension	37 (68.5)	30 (65.2)	
Amyloid angiopathy	2 (3.7)	4 (8.7)	
Aneurysm	3 (5.6)	0	
Arteriovenous malformation	5 (9.3)	0	
OAT in therapeutic range	3 (5.6)	6 (13.0)	
OAT out of therapeutic range	3 (5.6)	6 (13.0)	
Fibrinolytic treatment	1 (1.9)	0	
Intraventricular hemorrhage—n (%)	17 (31.5)	23 (50.0)	0.07
Hydrocephalus—n (%)	21 (38.9)	26 (56.5)	0.11
Evacuation SIH—n (%)	18 (33.3)	9 (19.6)	0.18
Age (years)—median (p 25–75)	59 (52–67)	68 (57–74)	0.006
Volume of SIH (cc)—median (p 25–75)	38 (17–62)	68 (29–99)	0.02
Midline shift (mm)—median (p 25–75)	1 (0–7)	5 (0–11)	0.005
Sodium (mEq/L)—median (p 25–75)	139 (137–142)	139 (135–143)	0.93
Temperature (°C)—median (p 25–75)	36.9 (36.0–37.4)	36.5 (35.0–37.0)	0.10
PaO2/FI0_2_ ratio—median (p 25–75)	270 (189–350)	289 (215–397)	0.40
Platelets—median*10^3^/mm^3^ (p 25–75)	193 (145–252)	198 (159–270)	0.57
INR—median (p 25–75)	1.10 (1.00–1.31)	1.14 (1.02–1.87)	0.34
Lactic acid (mmol/L)-median (p 25–75)	1.70 (1.00–2.51)	1.80 (1.30–2.55)	0.23
GCS score—median (p 25–75)	8 (6–8)	4 (3–6)	< 0.001
Glycemia (g/dL)—median (p 25–75)	141 (118–190)	170 (141–216)	0.01
aPTT (s)—median (p 25–75)	29 (27–32)	30 (24–34)	0.68
Fibrinogen (mg/dl)—median (p 25–75)	390 (280–493)	382 (350–510)	0.34
APACHE-II score—median (p 25–75)	18 (14–20)	24 (20–26)	<0.001
Creatinine (mg/dl)—median (p 25–75)	0.77 (0.68–0.90)	0.80 (0.60–1.01)	0.36
CCCK-18 (u/L)—median (p 25–75)	129 (112–177)	291 (212–341)	< 0.001

*P 25–75* 25th–75th percentile, *OAT* oral anticoagulant treatment, *PaO*_*2*_ pressure of arterial oxygen/fraction inspired oxygen, *FIO*_*2*_ pressure of arterial oxygen/fraction inspired oxygen, *INR* international normalized ratio, *GCS* Glasgow Coma Scale, *aPTT* activated partial thromboplastin time, *APACHE II* Acute Physiology and Chronic Health Evaluation, *CCCK* caspase-cleaved cytokeratin

We found the following correlations between serum CCCK-18 levels and age (rho = 0.19; p = 0.053), SIH volume (rho = 0.26; p = 0.02), midline shift (rho = 0.19; p = 0.10), GCS (rho = − 0.29; p = 0.003), APACHE-II score (rho = 0.32; p = 0.001). We have not found statistically significant differences in serum CCCK-18 levels in patients with or without intraventricular hemorrhage (p = 0.13), hydrocephalus (p = 0.11) or evacuation of SIH (p = 0.85).

**Fig. 1:**
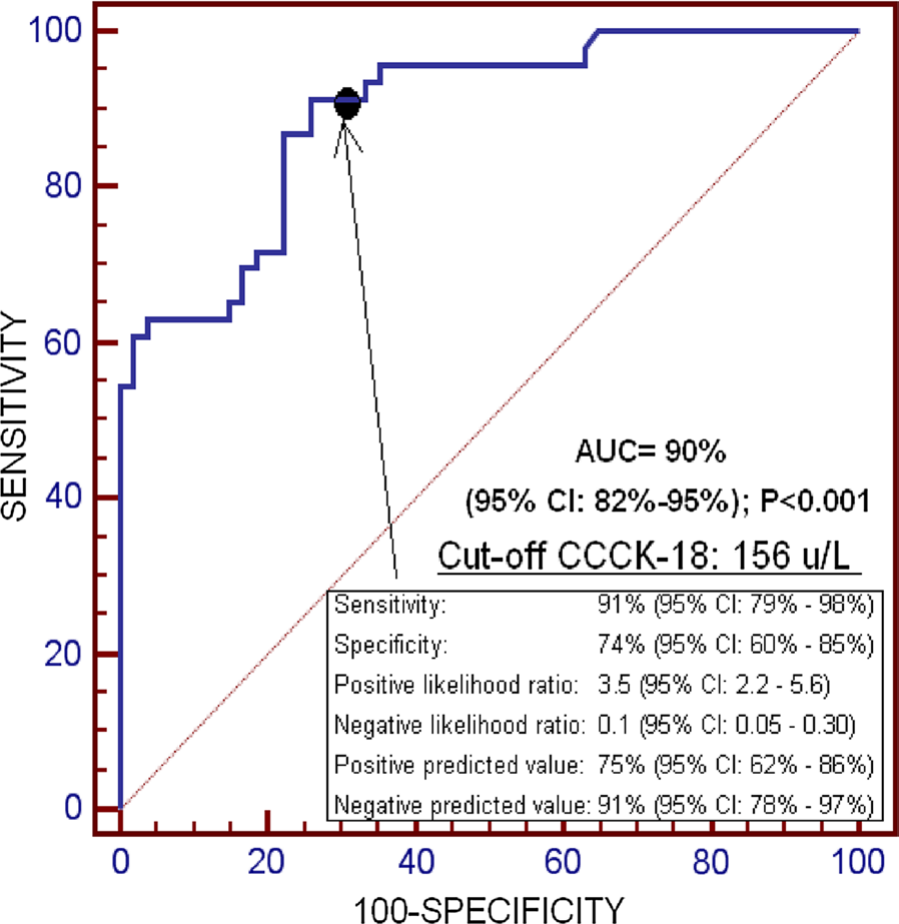
Receiver operation characteristic analysis using serum caspase-cleaved cytokeratin (CCCK)-18 levels as predictor of mortality at 30 days

We found the following area under the curve for 30-day mortality prediction: serum CCCK-18 levels of 90% (95% CI 82–95%; p < 0.001) (Fig. 1), age of 72% (95% CI 60–82%; p = 0.004), SIH volume of 68% (95% CI 56–79%; p = 0.02), midline shift of 68% (95% CI 55–78%; p = 0.003), GCS of 78% (95% CI 66–87%; p < 0.001), APACHE-II score of 81% (95% CI 69–89%; p < 0.001). We found the following differences in the comparisons in the area under the curve for 30-day mortality prediction between serum CCCK-18 levels and: age of 22% (95% CI 9–34%; p < 0.001), SIH volume of 25% (95% CI 12–39%; p < 0.001), midline shift of 26% (95% CI 12–40%; p < 0.001), GCS of 16% (95% CI 3–29%; p = 0.02), APACHE-II score of 13% (95% CI 1–26%; p = 0.046).

**Fig. 2:**
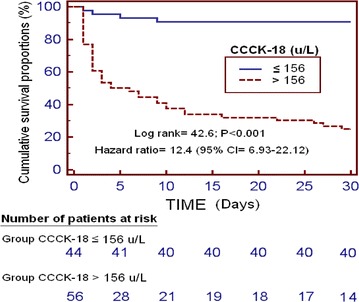
Survival curves at 30 days using serum levels of caspase-cleaved cytokeratin (CCCK)-18 higher or lower than 156 u/L

Kaplan–Meier 30-day survival analysis showed that patients with serum CCCK-18 levels higher than 156 u/L showed a higher mortality risk (Hazard ratio = 12.4; 95% CI 6.93–22.12; p < 0.001) (Fig. 2).

In the multiple logistic regression analysis, we found an association between serum CCCK-18 levels and 30-day mortality (OR = 1.034; 95% CI 1.013–1.055; p = 0.002) after to control for midline shift, GCS, SIH volume, intraventricular haemorrhage, and age (Table 2).

**Table 2:** Multiple logistic regression analysis to predict 30-day mortality

Variable	Odds ratio	95% confidence interval	*P*
Serum CCCK-18 levels (u/L)	1.034	1.013–1.055	0.002
Glasgow Coma Scale (points)	0.536	0.294–0.979	0.04
Midline shift (mm)	1.139	0.935–1.387	0.20
Age (years)	1.039	0.905–1.193	0.59
Volume of SIH (cc)	1.003	0.975–1.031	0.85
Intraventricular hemorrhage (yes)	1.136	0.133–9.727	0.91

*CCCK* caspase-cleaved cytokeratin

## Discussion

The novel finding of our study was that there is an association between high serum CCCK-18 levels and 30-day mortality in severe SIH patients.

Previously, higher circulating CCCK-18 levels have been associated with 6-month mortality in patients with basal ganglia hemorrhage [35]. However, the association between high serum CCCK-18 levels and early SIH mortality found in our study is a novel finding. There are some differences between the study by Gu et al. [35] and our study. In the study by Gu et al. patients were included with basal ganglia hemorrhage [35], and our study included patients with supratentorial SIH at different locations (lobar, basal ganglia, thalamus and periventricular). In our study, patients with SIH and GCS ≤ 8 were included, and in the study by Gu et al., the clinical condition of severity was not used for the inclusion of patients. In the study by Gu et al. the end-point study was late mortality (at 6 months), and in our study our end-point was early mortality (at 30 days). The findings of our study are in consonance with those of other previous studies by our team, of patients with traumatic brain injury, where we found an association between high serum CCCK-18 levels and 30-day mortality [33].

Our study presents some limitations. First, we do not report serum CCCK-18 concentrations in healthy controls; however, our objective study was to determine whether there is an association between serum CCCK-18 levels and SIH patient mortality and was not to determine whether SIH patients showed increased serum CCCK-18 levels. Second, there was not reported data about apoptosis in cerebral samples; however, our aim was to use an easy technique to facilitate the reproduction by other researchers. Third, data about the evolution of circulating CCCK-18 concentrations in non-surviving and surviving patients during the evolution were not reported.

CCCK-18 is not a specific biomarker of brain apoptosis; thus, higher circulating CCCK-18 levels have been found in patients with sepsis [21–25], liver diseases [26–30], and tumoral diseases [31, 32]. However, serum CCCK-18 levels have been associated with mortality in patients with SIH in the study by Gu et al. [35] and in our study. The clinical utility of serum CCCK-18 levels for mortality prediction should be taken with caution; however, we think that could be considered as an additional biomarker in the prognostic prediction of ICH patients to other markers (as GSC, age, midline shift, volume of SIH, or intraventricular hemorrhage). In addition, we found that serum CCCK-18 levels could be a better prognostic biomarker in ICH patients that age, SIH volume, midline shift, GCS, APACHE-II score according the area under the curve for 30-day mortality prediction of those variables.

Finally, the administration of antiapoptotic agents in SIH animal models have reduced cerebral apoptosis degree and functional deficits [9, 38–43]. Thus, all these findings could foster interest about research on apoptosis in SIH patients.

## Conclusions

The novel finding of our study was that there is an association between high serum CCCK-18 levels and 30-day mortality in severe SIH patients.

## Abbreviations

APACHE: Acute Physiology and Chronic Health Evaluation
CCCK: caspase-cleaved cytokeratin
FIO_2_: fraction inspired of oxygen
GCS: Glasgow Coma Scale
ICU: Intensive Care Unit
INR: international normalized ratio
ISS: Injury Severity Score
PaO_2_: pressure of arterial oxygen
